# Lung metastasis 21 years after initial diagnosis of osteosarcoma: a case report

**DOI:** 10.1186/1752-1947-3-9298

**Published:** 2009-11-24

**Authors:** Ari Halldorsson, Steven Brooks, Sam Montgomery, Suzanne Graham

**Affiliations:** 1 Division of Cardiothoracic Surgery, Department of Surgery, Texas Tech University, Lubbock, TX, USA; 2 Department of Pathology, Texas Tech University, Lubbock, TX, USA

## Abstract

**Introduction:**

To the best of our knowledge, this case report describes the longest disease-free interval between primary diagnosis and metastatic recurrence of an osteosarcoma.

**Case presentation:**

A 35-year-old Caucasian American man presented with asymptomatic lung metastases 21 years after being diagnosed and treated for lower extremity osteosarcoma. He underwent curative lung resection, but 2 years thereafter developed metastatic disease in the scapula and tibia and, after resection and chemotherapy, is in remission 1 year later.

**Conclusion:**

This case highlights the importance of long follow-up periods and continued surveillance of osteosarcoma patients after initial curative treatment.

## Introduction

Osteosarcoma is the third most common cancer in adolescence, after lymphomas and brain tumors. One-third of patients who present with osteosarcoma will have recurrent disease. Within the recurrent disease group, 95% of relapses occur within the first 5 years, with the lung as the most common site of metastases. Reported relapses beyond 10 years are rare in the literature. We describe the case of a 35-year-old man who presented with lung metastasis 21 years after undergoing surgical resection and adjuvant chemotherapy for osteosarcoma of the femur. The patient underwent a curative resection. Histological studies described this calcified lesion as osteosarcoma, thereby confirming, to the best of our knowledge, the longest known disease-free interval to metastatic recurrence reported in the English literature. We discuss the relevant clinical knowledge regarding late relapse of osteosarcoma.

## Case presentation

At the age of 14, the patient was diagnosed with osteosarcoma of the left femur. He received neoadjuvant chemotherapy consisting of doxorubicin and cisplatin, and underwent above-the-knee amputation of his left leg. Adjuvant chemotherapy included doxorubicin, methotrexate and vincristine.

**Figure 1:**
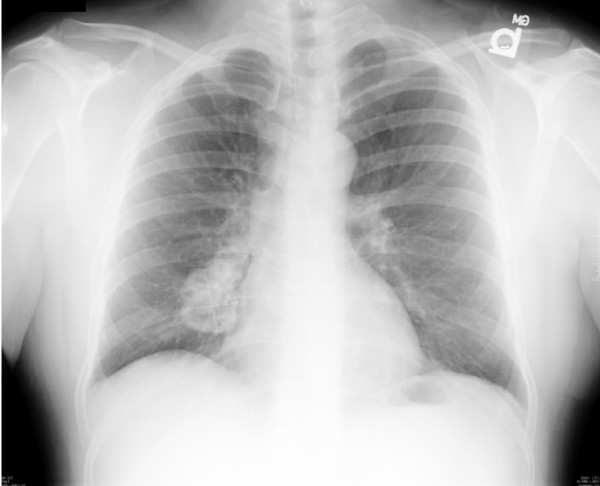
**Chest X-ray showing a right infrahilar tumor**.

**Figure 2:**
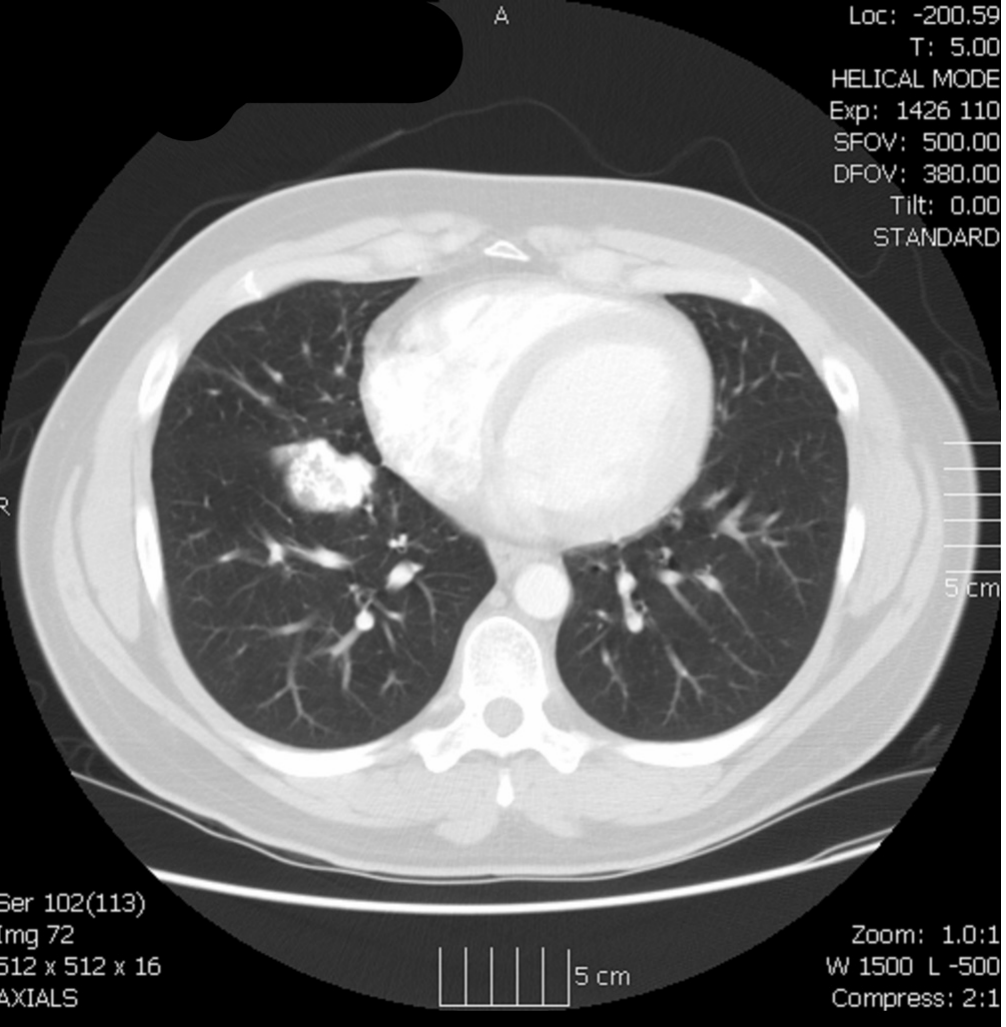
**Transaxial chest computed tomography scan demonstrating a calcified tumor in the right middle lobe adjacent to the oblique fissure**.

Twenty-one years after his initial osteosarcoma resection, the patient presented with cellulitis of his left thigh, which was treated successfully. During his hospital stay, a chest X-ray revealed a mass in the infrahilar area of the patient's right thorax (Figure 1). A computerized tomography (CT) scan described this 5 × 4 × 5 cm mass as considerably calcified with a Hounsfield unit density measurement identical to that of the patient's bone (Figure 2). There was no evidence of recurrence at the primary site and further metastatic work-up was negative.

**Figure 3:**
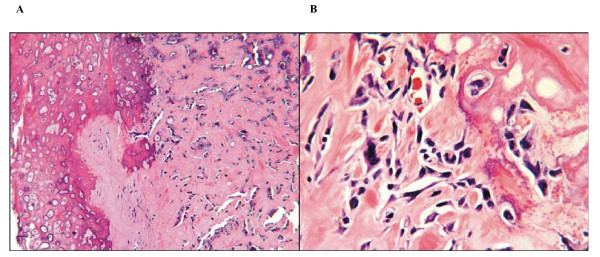
**A - Bony matrix predominates the lung mass with atypical cells trapped within**. Hemotoxylin and Eosin; original magnification 50×. **B - **Irregular hyperchromatic spindle cells within lesional matrix. Hematoxylin and Eosin; original magnification 125×.

The patient underwent curative resection, and pathology of the right middle and lower lung lobes revealed osteosarcoma with extensive chondroblastic differentiation (Figure 3a and 3b). This was consistent with the histologic description of the primary cancer. The patient was discharged within one week postoperatively and was well for two years postoperatively without signs of recurrence. Subsequently, the patient underwent surgical resection of the right tibia and partial resection of the left scapula for metastatic disease, after which he completed additional chemotherapy and is currently doing well three years after his lung surgery.

## Discussion

Osteosarcoma is the most common primary malignancy of the bone, with a peak incidence in the second decade and an additional peak of incidence in elderly individuals [1]. Historically, prognosis was abysmal, and although the addition of aggressive chemotherapeutic regiments to surgical excision has improved long-term survival, 30% to 40% of patients develop metastases [2], with the lung as the most common metastatic location [1,3-6]. It has been suggested that the addition of aggressive chemotherapy has altered the incidence, location and timing of metastasis [5]. Almost one-third of patients with osteosarcoma will experience recurrence of the disease. In these patients, 95% of relapses will occur within 5 years of the initial diagnosis, with an average time to relapse of 1.6 years [1,7]. The lung is the most common site of metastasis, with 77% to 92% of patients experiencing recurrence at this site [5], and an average survival after recurrence of less than 1 year [7]. Patients in a second remission have a 70% to 80% chance of second relapse within 1 year [6].

Late recurrence is usually described as occurring more than 5 years after primary osteosarcoma treatment. However, the recorded cases with the longest time spans before recurrence are all of local recurrence, such as the report by Lau *et al.*, which describes local recurrence 20 years after surgery [8]. The Rizzoli Institute study examined 648 patients from 1983 to 1997, with the longest local recurrence at 19.3 years. The longest time interval reported of metastatic recurrence in the Rizzoli study was 11.4 years. The Cooperative Osteosarcoma Study (COSS), in an analysis of 1,702 osteosarcoma patients treated between 1980 to 1998, described the longest time interval before recurrence at 14.3 years [8].

In 2004, Strauss and colleagues published an article describing eight patients seen at the London Bone and Soft tissue Tumor Service who had relapsed after long disease-free intervals [5]. The article also reviewed seven major studies on late relapse, defined as recurrence greater than 5 years after initial diagnosis, in patients treated for localized osteosarcoma. These seven studies included literature over a total of 23 years from Memorial Sloan Kettering, the Cooperative Osteosarcoma Study (COSS-86) analysis, three separate studies from the Rizzoli Institute, the Cooperative Osteosarcoma Study (COSS) intergroup analysis and Strauss' examination of 484 patients in London. Late relapse, defined as recurrence greater than 5 years after initial diagnosis, occurred in 57 out of 2,924 patients, an incidence of 1.9%. The longest disease-free interval to recurrence was the one previously mentioned from the COSS study at 14.3 years after treatment.

The most recent study on late recurrence comes from the Rizzoli Institute in 2006 and included 648 patients with non-metastatic osteosarcoma to determine predictive factors for late recurrence [4]. Twenty-four out of the 648 patients had late recurrent disease, and 20 out of the 24 patients who recurred had pulmonary metastases. The longest disease-free interval to metastatic recurrence was 11.4 years.

## Conclusion

Our patient was initially treated for osteosarcoma with surgical resection and chemotherapy, and presented 21 years later with a single metastasis to the right lung. Osteosarcomas are the least likely bone tumors to relapse late, making this case even more unusual. This case represents the longest disease-free interval to metastatic recurrence in the English literature, and underscores the need for continued surveillance of osteosarcoma patients.

## Competing interests

The authors declare that they have no competing interests.

## Authors' contributions

SG performed the histologic examination of the pathology specimens and was a major contributor in writing the manuscript. SB, SM and AH contributed significantly to the concept, design and writing of the manuscript. All authors read and approved the final manuscript.

## Consent

Written informed consent was obtained from the patient for publication of this case report and any accompanying images. A copy of the written consent is available for review by the Editor-in-Chief of this journal.
